# Hot Deformation Behavior and Processing Maps of an As-Cast Al-5Mg-3Zn-1Cu (wt%) Alloy

**DOI:** 10.3390/ma16114093

**Published:** 2023-05-31

**Authors:** Chuan Lei, Qudong Wang, Mahmoud Ebrahimi, Dezhi Li, Huaping Tang, Nannan Zhang, Huisheng Cai

**Affiliations:** 1 National Engineering Research Center of Light Alloy Net Forming, State Key Laboratory of Metal Matrix Composites, School of Materials Science and Engineering, Shanghai Jiao Tong University, Shanghai 200240, China; leichuan@sjtu.edu.cn (C.L.); ebrahimi@maragheh.ac.ir (M.E.); jszhangnan@126.com (N.Z.); caihuisheng@sjtu.edu.cn (H.C.); 2 Warwick Manufacturing Group, University of Warwick, Coventry CV4 7AL, UK; 3 Ji Hua Laboratory, Foshan 528255, China; tanghp@jihualab.com

**Keywords:** Al-Mg-Zn-Cu crossover alloy, hot deformation, constitutive analysis, processing maps, microstructural evolution

## Abstract

One of the key issues limiting the application of Al-Mg-Zn-Cu alloys in the automotive industry is forming at a low cost. Isothermal uniaxial compression was accomplished in the range of 300–450 °C, 0.001–10 s^−1^ to study the hot deformation behavior of an as-cast Al-5.07Mg-3.01Zn-1.11Cu-0.01Ti alloy. Its rheological behavior presented characteristics of work-hardening followed by dynamic softening and its flow stress was accurately described by the proposed strain-compensated Arrhenius-type constitutive model. Three-dimensional processing maps were established. The instability was mainly concentrated in regions with high strain rates or low temperatures, with cracking being the main instability. A workable domain was determined as 385–450 °C, 0.001–0.26 s^−1^, in which dynamic recovery (DRV) and dynamic recrystallization (DRX) occurred. As the temperature rose, the dominant dynamic softening mechanism shifted from DRV to DRX. The DRX mechanisms transformed from continuous dynamic recrystallization (CDRX), discontinuous dynamic recrystallization (DDRX), and particle-stimulated nucleation (PSN) at 350 °C, 0.1 s^−1^ to CDRX and DDRX at 450 °C, 0.01 s^−1^, and eventually to DDRX at 450 °C, 0.001 s^−1^. The eutectic T-Mg_32_(AlZnCu)_49_ phase facilitated DRX nucleation and did not trigger instability in the workable domain. This work demonstrates that the workability of as-cast Al-Mg-Zn-Cu alloys with low Zn/Mg ratios is sufficient for hot forming.

## 1. Introduction

Aluminum alloys have been the preferred candidates for lightweighting in aerospace, transportation, and other sectors due to their high specific strength [1,2,3,4]. However, traditional commercial aluminum alloys gradually fail to satisfy the increasingly stringent requirements of mechanical properties and corrosion resistance, etc. [1,5]. Therefore, it is urgent to develop aluminum alloys with a better comprehensive performance. Recently, Al-Mg-Zn-Cu alloys with low Zn/Mg ratios (<1, also conceptualized as crossover alloys) have received particular attention [6,7,8,9]. By optimizing their compositions, for instance, adjusting the contents and proportions of the Zn, Mg, and Cu elements [7,10], or introducing microalloying elements such as Si and Ag [11,12], a large amount of T-Mg_32_(Al,Zn)_49_ type precipitates are introduced into the alloys to ensure a high strength [7,9,11]. By implementing an appropriate heat treatment, such as two-stage aging or a thermomechanical treatment, the distribution of the precipitates can be regulated, thereby improving the mechanical properties and corrosion resistance [6,9,13]. In addition, plastic deformation can also be implemented to further improve the mechanical properties of these alloys [14,15]. Additionally, research has shown that these alloys have a good adaptability to casting, plastic deformation, and additive manufacturing [8,16,17], and have a satisfactory weldability [18]. These excellent characteristics indicate that Al-Mg-Zn-Cu alloys with low Zn/Mg ratios have promising application prospects in the automotive industry [6,7]. However, there is still a lack of research on their formability, although this is important.

Generally, Al-Mg-Zn-Cu alloys are formed by hot working, so it is particularly important to have a comprehensive understanding of hot workability [19,20,21], which is mainly reflected in three objectives: (1) understanding the evolution of stress–strain, (2) formulating processing strategies, and (3) regulating the microstructure [22,23,24]. The rheological behavior can be described by appropriate constitutive models; hence, a variety of constitutive models have been developed, including phenomenological, physics-based, and statistical models [3,4,25]. Phenomenological constitutive models are used widely because of their conciseness and high accuracy [22,26]. Zhao et al. [26] established an Arrhenius-type constitutive model for an Al-5.5Zn-2.2Mg-2.1Cu alloy, which can accurately predict flow stress (*σ*). Processing maps have been utilized broadly in formulating processing strategies [19,21]. Raja et al. [19] and Wang et al. [21] sequentially determined the workable domains of Al-7.3Zn-2.2Mg-2Cu and Al-10Zn-3Mg-2.8Cu alloys based on processing maps. Additionally, Al-Mg-Zn-Cu alloys undergo a complex microstructure evolution during hot deformation, including DRV, DRX, and precipitation, etc. [20,25,26,27]. Multiple DRXs may occur during the hot working of Al alloys, including continuous dynamic recrystallization (CDRX), discontinuous dynamic recrystallization (DDRX), and geometric dynamic recrystallization (GDRX) [19,28,29]. Furthermore, there may also be the formation or dissolution of precipitates or second phases [30]. However, research on the hot workability of Al-Mg-Zn-Cu alloys with low Zn/Mg ratios is scarce. Lei et al. [17] and Khomutov et al. [31] modeled the flow behavior of Al-5Mg-3Zn-1Cu and Al-4.5Mg-4.5Zn-1Cu-Sc alloys, respectively, and determined the corresponding hot-working window based on processing maps, but a systematic study on the microstructure evolution was lacking, and these studies were limited to homogenized alloys only.

Homogenization is usually introduced into process chains to improve the formability of Al-Mg-Zn-Cu alloys, but this procedure increases the energy consumption and equipment investment and reduces the production efficiency [32,33]. For this reason, some researchers prefer to process the cast slab directly and have developed a series of processing techniques to do this, such as continuous casting and rolling, casting–forging hybrid forming, and casting/spinning forming, etc. [1,5,34]. Although these techniques pose more severe challenges to the formability of as-cast alloys, several studies have emphasized the feasibility and superiority of the direct hot working of as-cast Al-Mg-Zn-Cu alloys [33,35,36,37]. Guo et al. [33] and Geong et al. [38] demonstrated that the hot workability of as-cast Al-Mg-Zn-Cu alloys is also acceptable, even better than that of homogenized alloys [35,36]. Moreover, it has been proved that, via the direct hot forming of an unhomogenized blank, the forming speed was accelerated [37] and the mechanical properties and anisotropy of the alloys were improved [37,39]. Nevertheless, the hot workability of Al-Mg-Zn-Cu alloys with low Zn/Mg ratios has not been sufficiently investigated.

This work was dedicated to exploring the adaptability of as-cast Al-5Mg-3Zn-1Cu alloys to hot forming. In this regard, hot flow curves were obtained using uniaxial compression, accordingly, an Arrhenius-type constitutive model for flow stress was established, and the model was correlated with strain by establishing compensation equations. Three-dimensional processing maps were established to determine the appropriate workability interval. The evolution of grains, eutectic phases, and precipitates during hot deformation was studied, with a focus on exploring the DRX mechanisms and the relevant evolution with deformation parameters. This study is expected to correlate flow behavior, hot workability, and the microstructure to provide a theoretical basis for addressing the problem of the hot forming of as-cast Al-Mg-Zn-Cu alloys with low Zn/Mg ratios.

## 2. Materials and Methods

The alloy used in this study was prepared using permanent mold gravity casting, and commercial-purity Al, Mg, Zn, and Al-50Cu (wt%) master alloys were melted in an electric resistance furnace. Degassing was performed with C_2_Cl_6_ powder after the melt was stabilized at 720 °C. After skimming off the dross, a 0.2 wt% Al-5Ti-1B master alloy was added to the melt for grain refinement. After 5 min of incubation, the melt was poured into a permanent mold that had been preheated to 100 °C. The chemical composition of the alloy was measured using an inductively coupled plasma atomic emission spectrometer, and the actual composition of the alloy was Al-5.07Mg-3.01Zn-1.11Cu-0.01Ti (wt%).

Samples with diameters and heights of 8 and 12 mm, respectively, were machined from the ingots. Isothermal compression was implemented on a Gleeble-3800 thermo-mechanical simulation system and the range of the deformation parameters selected was 300–450 °C, 0.001–10 s^−1^. The samples were heated to the experimental temperature (*T*) at 5 °C/s and held for 3 min, and then compressed to 0.9 true strain (*ε*) at the designated strain rates ($\dot{\varepsilon }$). The temperature of the samples was monitored using thermocouples welded to their surface. After compression, water was promptly sprayed onto the squashed samples to maximize their retention of the deformed microstructure.

The compressed specimens turned into a barrel shape, the bulging regions of the samples were meticulously inspected for instability identification, then the microstructures of the central regions of the samples were analyzed. The sections for the scanning electron microscopy (SEM) analysis were mechanically polished with MgO suspension, and energy dispersive X-ray spectroscopy (EDS) was utilized for a semi-quantitative composition analysis of the eutectic phases. Additionally, partial mechanically polished samples were further polished using colloidal silica suspension for an electron backscatter diffraction (EBSD) analysis; the step size for the EBSD test was 1.5 μm and the EBSD data were post-processed using the Channel 5 package.

## 3. Results and Discussion

### 3.1. Undeformed Microstructure

**Figure 1:**
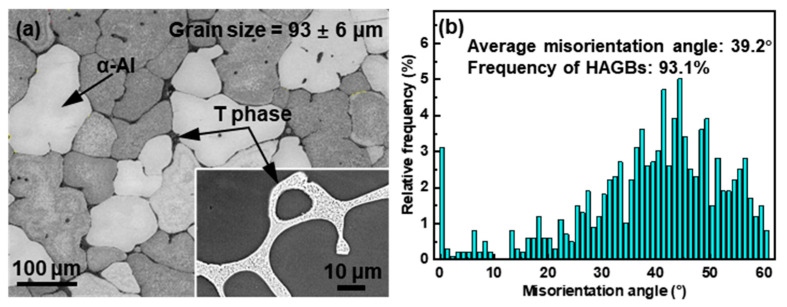
Microstructure of the undeformed alloy: (**a**) EBSD band contrast map (the insert is the magnified microstructure of the eutectics), and (**b**) corresponding misorientation angle distribution.

Figure 1 presents the undeformed microstructure of the alloy. The α-Al presented an equiaxed dendritic morphology and the average grain size was 93 ± 6 μm, as shown in Figure 1a. The average misorientation angle was 39.2° and most of the grain boundaries (93.1%) were high-angle grain boundaries (HAGBs, >15°), as shown in Figure 1b. The insert in Figure 1a shows the reticular eutectic phase at the grain boundaries. Their composition was Mg: 29.8 at.%, Al: 54.7 at.%, Zn: 9.7 at.%, and Cu: 5.8 at.%. Previous studies have confirmed that the eutectic phase was T-Mg_32_(AlZnCu)_49_ phase (labeled as T phase for brevity) [8].

### 3.2. Hot Compression Curves

**Figure 2:**
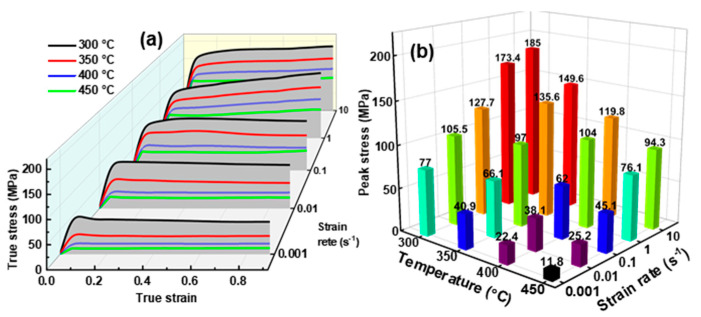
(**a**) Hot compression curves, and (**b**) corresponding peak stress of the alloy.

During the thermal simulation experiment, the friction between the samples with the die, as well as the heat generation inside the samples, caused the obtained flow stress to deviate from the true value at the set temperature. In this work, a friction correction and temperature correction were performed on the measured flow stress using the methods in Ref. [40]. Figure 2a shows the corrected hot compression curves of the alloy, and the corresponding peak stress is illustrated in Figure 2b. The flow curves embody the confrontation between work-hardening and softening. At the onset of the compression, a slight increase in the *ε* caused a dramatic elevation of *σ* to the peak stress, reflecting the swift work-hardening associated with the rapid dislocation proliferation [19]. The peak stress increased from 11.8 MPa to 185 MPa with a simultaneous increase in the *T* and $\dot{\varepsilon }$, reflecting a sharp increase in the deformation resistance. As the deformation proceeded, dynamic softening gradually prevailed, and the *σ* stabilized after a transitional stage. Generally, dynamic softening is subdivided into DRV and DRX, depending on the stabilizing stresses [28]. As shown in Figure 2a, some curves were stable at the peak stress, indicating that only DRV occurred, while the stable stress of the other curves was lower than the peak stress, indicating that DRX occurred [27].

### 3.3. Constitutive Model

#### 3.3.1. Constitutive Parameters

Based on the Arrhenius creep framework, the correlation of $\dot{\varepsilon }$, *σ*, and *T* at high temperatures is as follows [20,21]:

(1) $$\dot{\varepsilon }=Af\left(\sigma \right)exp\left(-Q/RT\right)$$

where *A* denotes a material parameter, *Q* is the deformation activation energy, *R* represents the universal gas constant, and *R =* 8.314 J/(mol·K).

The *σ* sequentially follows the power, exponential, and hyperbolic-sine laws at low, high, and all stress levels, respectively [4]:

(2) $$f\left(\sigma \right)=\left\{\begin{array}{c} \sigma^{n_{1}}(\alpha \sigma <0.8)\\ \exp\left(\beta \sigma \right)(\alpha \sigma >1.2)\\ {\left[sinh\left(\alpha \sigma \right)\right]}^{n}(all\;stress\;level) \end{array}\right.$$

where *n*_1_, *α*, *β*, and *n* are material constants and *β* = *αn*_1_. Substituting Equation (2) into Equation (1) and converting it into logarithmic form obtains:

(3) $$ln\dot{\varepsilon }=\left\{\begin{array}{c} \ln A_{1}+n_{1}\ln\sigma -Q/RT\left(\alpha \sigma <0.8\right)\\ \ln A_{2}+\beta \sigma -Q/RT\left(\alpha \sigma >1.2\right)\\ \ln A+n\ln\left[sinh\left(\alpha \sigma \right)\right]-Q/RT\left(all\;stress\;level\right) \end{array}\right.$$

where *A*_1_ and *A*_2_ are constants.

*Q* is extracted by differentiating the third sub-item of Equation (3):

(4) $$Q=R{\left\{\frac{\partial ln\left[sinh\left(\alpha \sigma \right)\right]}{\partial \left(1/T\right)}\right\}}_{\dot{\varepsilon }}{\left\{\frac{ln\dot{\varepsilon }}{\partial ln\left[sinh\left(\alpha \sigma \right)\right]}\right\}}_{T}$$

The Zener–Hollomon parameter ($Z=\dot{\varepsilon }exp\left(Q/RT\right)$) is introduced to incarnate the synergy of $\dot{\varepsilon }$ and *T* [23,41], and the third sub-item of Equation (3) is converted into:

(5) $$lnZ=lnA+nln\left[sinh\left(\alpha \sigma \right)\right]$$

**Figure 3:**
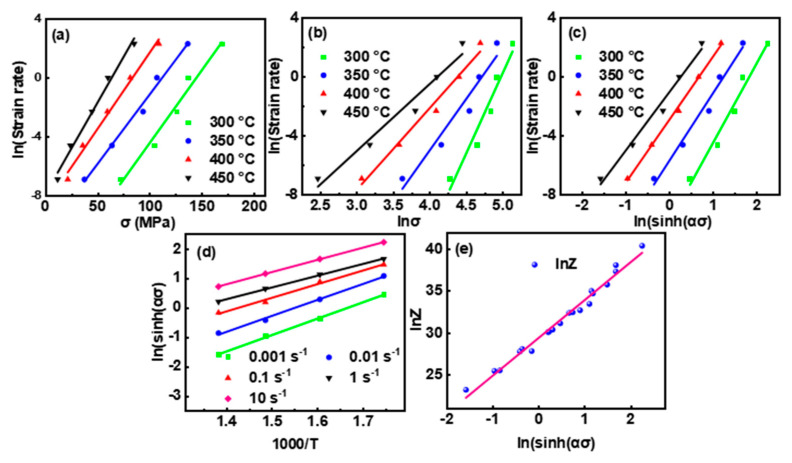
Linear fitting of (a) $ln\dot{\varepsilon }$ − *σ*, (**b**) $ln\dot{\varepsilon }$ − ln*σ*, (**c**) $ln\dot{\varepsilon }$ − ln[sinh(*ασ*)], (**d**) ln[sinh(*ασ*)] − 1000/*T*, and (**e**) ln*Z* − ln[sinh(*ασ*)] at *ε* = 0.2.

Based on Equations (3)–(5), Figure 3 sequentially presents the correlations between ln$\dot{\varepsilon }$, *σ*, ln*σ*, ln[sinh(*ασ*)], 1000/*T,* and ln*Z* at *ε* = 0.2. Linear fitting was applied to extract the pending parameters by calculating the arithmetic means of the slopes of the fitting curves in Figure 3a–d. The exact values of *β*, *n*_1_, *α,* and *n* were 0.099, 5.704, 0.017, and 4.58, respectively, while *Q* was 181.6 kJ/mol. ln*A* was extracted to be 29.5 by calculating the intercept of the fitting curve in Figure 3e.

The derived constitutive parameters *n* can be used to speculate on the movement modes of the dislocations or grain boundaries [2,19,35]. At *ε* = 0.2, the calculated *n* were, successively, 5.42, 4.61, 4.30, and 3.99 at 300 °C, 350 °C, 400 °C, and 450 °C. *n* was close to 5 at 300 °C, according to Refs. [2,42], and the predominant deformation mechanisms were dislocation gliding and climbing. *n* decreased with increasing *T*, indicating a decrease in the barrier of dislocation or boundary migration, which is usually associated with the promoted grain boundary sliding, DRX, and inhibited precipitation at high temperatures [3].

#### 3.3.2. Strain Compensation

**Figure 4:**
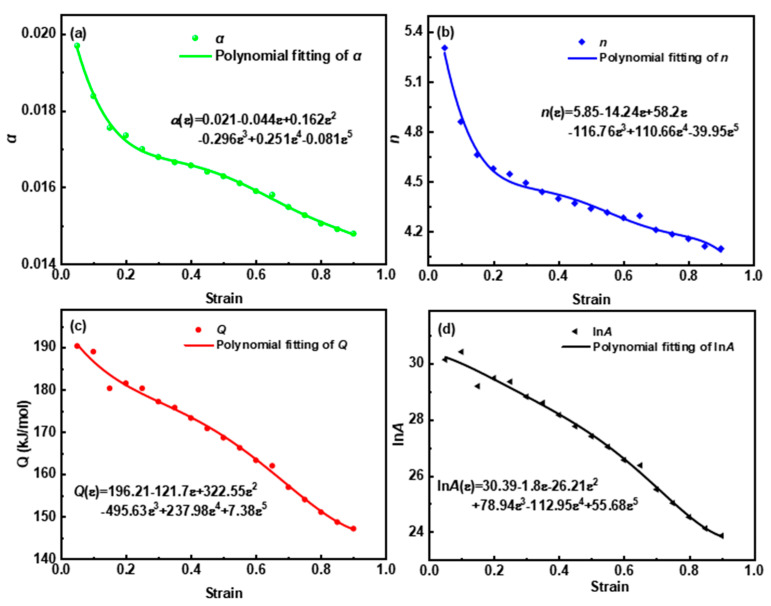
Values and associated quintic polynomial fitting curves of (**a**) *α*, (**b**) *n*, (**c**) *Q,* and (**d**) ln*A*.

To reflect the strain dependence of the constitutive model, the constitutive parameters at other strains were derived and the compensation functions were established using quintic polynomial fitting [25,26]. As shown in Figure 4, the compensation functions accurately reflected the evolution of the parameters, except for the small fluctuations of *Q* and ln*A* when *ε* < 0.2. Accordingly, the strain-compensated constitutive model applicable to *ε* = 0–0.9 is:

(6) $$\left\{\begin{array}{c} \sigma =ln\left\{{\left(Z/A\left(\varepsilon \right)\right)}^{1/n\left(\varepsilon \right)}+{\left[{\left(Z/A\left(\varepsilon \right)\right)}^{2/n\left(\varepsilon \right)}+1\right]}^{1/2}\right\}/\alpha \left(\varepsilon \right)\\ \alpha \left(\varepsilon \right)=0.021-0.044\varepsilon +0.162\varepsilon^{2}-0.296\varepsilon^{3}+0.25\varepsilon^{4}-0.081\varepsilon^{5}\\ n\left(\varepsilon \right)=5.85-14.24\varepsilon +58.2\varepsilon^{2}-116.76\varepsilon^{3}+110.66\varepsilon^{4}-39.95\varepsilon^{5}\\ Q\left(\varepsilon \right)=196.21-121.7\varepsilon +322.55\varepsilon^{2}-495.63\varepsilon^{3}+237.98\varepsilon^{4}+7.38\varepsilon^{5}\\ lnA\left(\varepsilon \right)=60.39-1.8\varepsilon -26.21\varepsilon^{2}+78.94\varepsilon^{3}-112.95\varepsilon^{4}+55.68\varepsilon^{5} \end{array}\right.$$

#### 3.3.3. Accuracy of the Model

**Figure 5:**
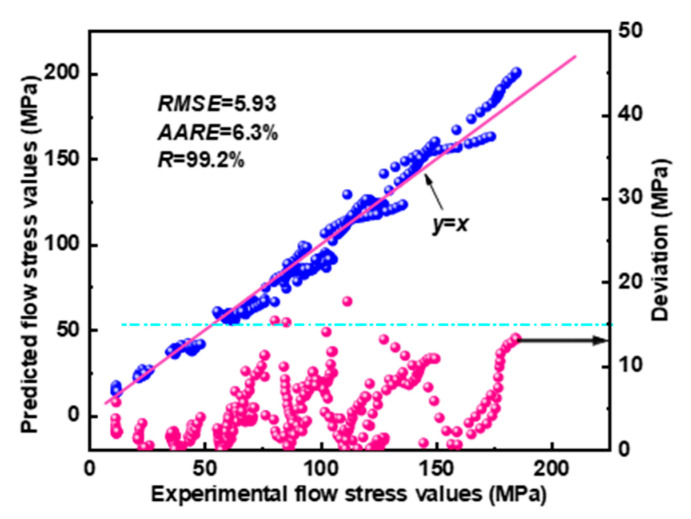
Correlation of the experimental and predicted flow stress.

The experimental and predicted flow stress were compared and the deviation between them was calculated. Furthermore, three statistical parameters (Equations (7)–(9)), namely the root mean square error (*RMSE*), average absolute relative error (*AARE*), and correlation coefficient (*R*), were calculated [4,25]. As demonstrated in Figure 5, the corresponding predicted and experimental values were distributed near the y = x curve and most of the deviation values were less than 15 MPa. *RMSE* and *AARE* were only 5.93 and 6.3%, respectively, and *R* reached 99.2%, demonstrating the satisfactory accuracy of the constitutive model.

(7) $$RMSE=\sqrt{\left[\sum_{i=1}^{N}{\left(E_{i}-P_{i}\right)}^{2}\right]/N}$$

(8) $$AARE\left(\%\right)=\left[\sum_{i=1}^{N}\left|\left(E_{i}-P_{i}\right)/E_{i}\right|\right]/N\times 100$$

(9) $$R=\left[\sum_{i=1}^{N}\left(E_{i}-\overset{-}{E})(P_{i}-\overset{-}{P}\right)\right]/\sqrt{\sum_{i=1}^{N}{\left(E_{i}-\overset{-}{E}\right)}^{2}\sum_{i=1}^{N}{\left(P_{i}-\overset{-}{P}\right)}^{2}}$$

### 3.4. Processing Maps

Processing maps were constructed based on the dynamic material model, which treats metal processing as an irreversible power dissipation system containing source, storage, and consumption units [24,43]. During hot working, the power dissipation importation (*P*) satisfies:

(10) P=G+J

where *G =* $\int_{0}^{\dot{\varepsilon }}\sigma d\dot{\varepsilon }$ and $J=\int_{0}^{\sigma }\dot{\varepsilon }d\sigma$ embody the power dissipation induced by the plastic deformation and microstructural evolution successively. Parameter *m* (the strain rate sensitivity) is defined as the partition between *G* and *J* [24]:

(11) $$m=\frac{dJ}{dG}=\frac{\partial P}{\partial G}\frac{\partial J}{\partial P}=\frac{\dot{\varepsilon }d\sigma }{\sigma d\dot{\varepsilon }}=\frac{\partial \left(ln\sigma \right)}{\partial \left(ln\dot{\varepsilon }\right)}$$

Assuming that the processing follows the power law [43]:

(12) $$\sigma =K{\dot{\varepsilon }}^{m}$$

With *K* as a material-dependent coefficient. *J* is modified to:

(13) $$J=m\sigma {\dot{\varepsilon }}^{m}/\left(m+1\right){|}_{\varepsilon ,T}$$

If *J* reaches the theoretical maximum value of *J*_max_ = *P*/2 in ideal plastic flow (*m* = 1), then the power dissipation coefficient is defined as the proportion of *J* to *J*_max_ [36]:

(14) $$\eta =J/J_{max}=\left[m\sigma \dot{\varepsilon }/\left(m+1\right)\right]/\left(\sigma \dot{\varepsilon }/2\right)=2m/\left(m+1\right)$$

On the other hand, the flow instability is identified by the criteria [38,43]:

(15) $$\xi =\frac{\partial ln\left[m/\left(m+1\right)\right]}{\partial \left(ln\dot{\varepsilon }\right)}+m<0$$

A negative *ξ* indicates flow instabilities, which are usually manifested as crack, flow localization, adiabatic shear band, and void, etc. [43].

**Figure 6:**
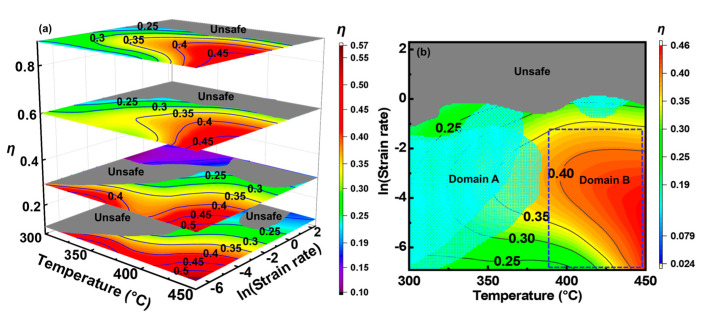
(**a**) 3D processing map of the alloy, and (**b**) superimposed processing maps at different strains.

Figure 6a shows the 3D processing maps of the alloy. The representative *η* values (in percent) are labeled on the contour lines and the unsafe zones are rendered in grey. The *η* generally increased with an increasing temperature and decreasing $\dot{\varepsilon }$, but decreased with an increasing strain. Instability generally occurred in regions where *η* < 0.25. There were two unsafe domains at a 0.1 strain, with corresponding deformation parameters of 395–433 °C, 0.47–10 s^−1^ and 300–383 °C, 0.001–0.34 s^−1^, respectively. The two unsafe domains coalesced when the strain reached 0.3 and gradually encroached on the entire scope of $\dot{\varepsilon }$ > 0.42 as the strain increased. To reflect the integral effect of the strain, the processing maps under different strains were superimposed and are shown in Figure 6b. The grid region (domain A) marks the region where the safety was uncertain under different strains. Although this domain owned a moderate *η* (0.25–0.36), which is the range where DRV and even DRX may occur [36], the confusion of the instability factors indicates that the deformation safety was not guaranteed [44].

In contrast, the range of 385–450 °C and 0.001–0.26 s^−1^ (domain B) always belongs to the safe zone. In domain B, the *η* exceeded 0.37 under all the strains, indicating that the deformation energy storage was consumed efficiently by microstructural changes, most likely DRX [39]. Therefore, the appropriate processable domain should be 385–450 °C, 0.001–0.26 s^−1^.

**Figure 7:**
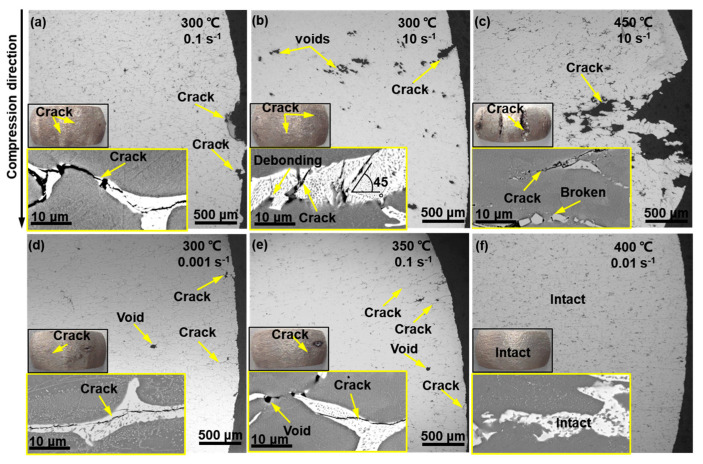
Macroscopic and microscopic morphology of the alloy compressed in (**a**–**c**) unsafe domain, (**d**,**e**) domain A, and (**f**) safe domain, the insets show the SEM images of the crack tips.

As shown in Figure 7, the samples were dissected to verify the reliability of the processing maps. The samples compressed in the unsafe domains (Figure 7a–c) were cracked, especially at 450 °C, 10 s^−1^. The metallographic photographs demonstrate that the cracks originated from the bulge regions, and dense voids can also be observed near these cracks. A similar cracking situation was also observed for the samples deformed in domain A (Figure 7d,e), indicating that this instability also occurred in the moderate *η* domain. Conversely, the sample deformed in the safe zone was intact, (Figure 7f). The above observations are highly consistent with the established processing maps.

At the crack tips (insets in Figure 7), debonding between the matrix and eutectic T phase and the fracture of the eutectics phase were observed, indicating that the eutectic T phase facilitated the crack propagation. In the unsafe zone, the brittle eutectic T phases had neither sufficient deformation ability nor the ability to coordinate matrix deformation, causing stress concentration and deteriorating the workability [33]. The cracking was especially severe at high temperatures and high strain rates. In this case, the bonding strength between the grains was severely weakened due to the adiabatic-temperature-rise-induced weakening of the grain boundary phase [45]. At 450 °C, 10 s^−1^, the deformation-induced temperature rise, ∆*T,* is quantified as ∆*T* = *γH/C*, where *γ* = 0.97 is the heat transformation efficiency, *H =* ∫σdε is the strain energy of the plastic deformation, and *C* = *ρC_p_* is the heat capacity (*J*/°C), in which *ρ* = 2.6 g/cm^3^ and *C_p_* = 1.24 J/(g·K) represent the density and specific heat of the alloy at 450 °C. As a result, ∆*T* reached 26.3 °C, implying that the actual temperature of the sample during the deformation approached the incipient melting point of the eutectic T phase [31], thereby inducing cracks with the assistance of strain. However, no eutectic-induced instability was observed in the safe zone. Similar studies have also shown that the as-cast 7075 alloy is less prone to flow instability than the homogenized alloy [35]. When deformed in the safe zone, the eutectic T phase underwent plastic deformation to coordinate the deformation of the matrix, as also observed for the 7075 and Al-7.93Zn-2.68Mg-2.0Cu alloys [35,36]. Such an excellent deformation compatibility of the eutectic T phase and α-Al matrix can be attributed to the unique high-temperature nature of the T phase and favorable interfacial adaptation of α-Al and the T phase [46]. In this case, the stress concentration at the α-Al/T phase interfaces was insufficient for crack initiation.

### 3.5. Microstructural Evolution and Dynamic Softening Mechanism

#### 3.5.1. Effect of Deformation Parameters on Secondary Phases

**Figure 8:**
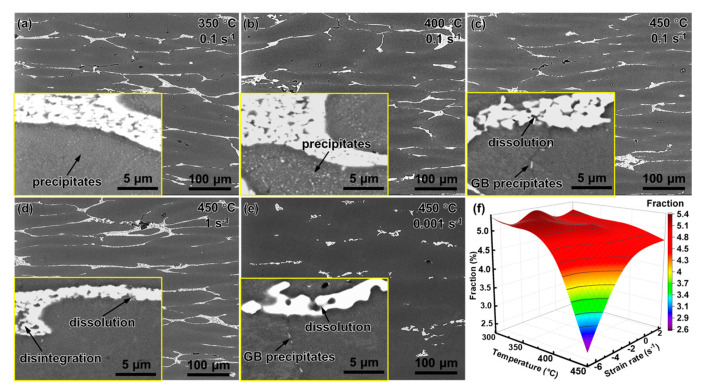
SEM images of the alloy after compression: (**a**) 350 °C, 0.1 s^−1^, (**b**) 400 °C, 0.1 s^−1^, (**c**) 450 °C, 0.1 s^−1^, (**d**) 450 °C, 1 s^−1^, (**e**) 450 °C, 0.001 s^−1^, and (**f**) correlation between the fraction of eutectic T phase and temperature and strain rate.

Figure 8a–e show the SEM images of the alloy deformed at various parameters. The content of the eutectic T phase changed little below 400 °C, but significantly decreased at 450 °C. The diffusion coefficient is quantified as:

(16) $$D=D_{0}exp\left(-Q/RT\right)$$

where *D*_0_ is the diffusion constant and *Q* is the diffusion activation energy of the solute. According to Equation (16), as the temperature increased, the diffusion coefficient increased sharply [8]. When the temperature was below 400 °C, the diffusion coefficient was low and the reduction in the eutectic phase caused by the diffusion was negligible. When the temperature reached 450 °C, the diffusion coefficient increased and the solute in the eutectic phase dissolved rapidly into the matrix. Figure 8f shows the statistics of the fraction of the eutectic T phase at 450 °C, the dissolution of the eutectic T phase intensifying as the strain rate decreases, and the fraction of the eutectic T phase in the sample deforming at 450 °C, 0.001 s^−1^, decreasing to 45% of that before the deformation. It was shown that dislocation-assisted diffusion promoted the dissolution of the eutectic T phase [47]. At a high temperature, the generation/movement of both dislocations and vacancies was promoted, which significantly accelerated the solute diffusion [47].

The illustration in Figure 8a–e shows the evolution of the dynamic precipitates. At 350 °C, intragranular and intergranular precipitates coexisted (Figure 8a). The quantity density of the precipitates was high because the driving force for precipitation at low temperatures is strong, and a large number of dislocations provides abundant nucleation sites [20]. The precipitates coarsened with an increase in the temperature to 400 °C (Figure 8b). However, when the temperature continued to rise to 450 °C, the intragranular precipitates disappeared and the grain boundary precipitates were also significantly coarsened and greatly reduced. Previous studies have shown that dynamic precipitates are relevant to the high apparent activation energy of highly alloyed Al-Mg-Zn-Cu alloys. In this study, *Q* was always higher than the lattice diffusion activation energy of aluminum (142 kJ/mol), implying that the deformation was hindered by supplementary impediments, apart from high-temperature diffusion [20,30,48]. The additional activation energy is most likely attributed to the dislocation pinning induced by solutes and precipitates [31]. These fine and dispersed precipitates may pin grain boundaries and dislocations, thereby inhibiting the process of grain evolution [19].

#### 3.5.2. Effect of Deformation Parameters on Grain Structure

**Figure 9:**
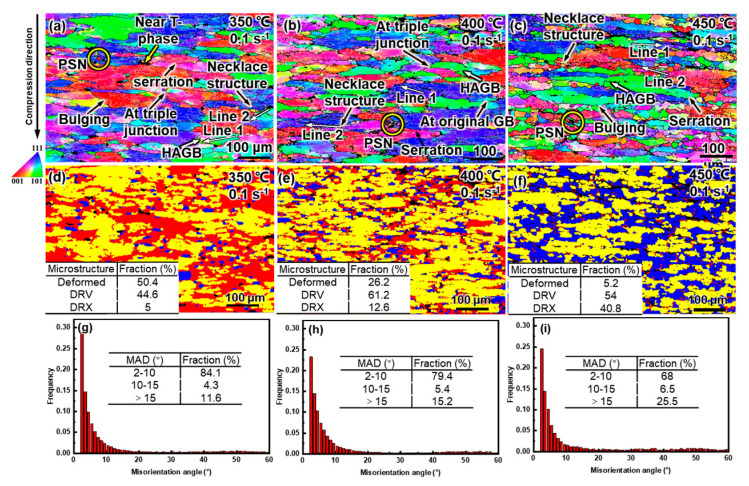
IPF maps (**a**–**c**), DRX maps (**d**–**f**), and MADs (**g**–**i**) of the alloy after compression at 0.1 s^−1^ at: (**a**,**d**,**g**) 350 °C, (**b**,**e**,**h**) 400 °C, and (**c**,**f**,**i**) 450 °C.

Figure 9 shows the microstructures of the alloy compressed at different temperatures at 0.1 s^−1^. As illustrated in the IPF maps in Figure 9a–c, the grains were flattened and awash with abundant low-angle grain boundaries (LAGBs, 2–10°), and orientation gradients commonly existed. The microstructure distribution is shown in Figure 9d–f; the DRX, DRV, and deformed grains are sequentially rendered in blue, yellow, and red. At 350 °C, most of the grains were either in deformed (50.4%) or DRV (44.6%) states, and with an increase in temperature to 450 °C, the proportion of the deformed grains decreased rapidly to 5.2%, but the proportion of DRX increased sharply to 40.8% and the proportion of DRV increased first and then decreased. The evolution of the misorientation angle distributions (MADs) was consistent with the microstructure proportion (Figure 9g–i). As the temperature increased from 350 °C to 450 °C, the proportion of LAGBs gradually decreased from 84.1% to 68%, while the proportion of HAGBs gradually increased from 11.6% to 25.5%.

**Figure 10:**
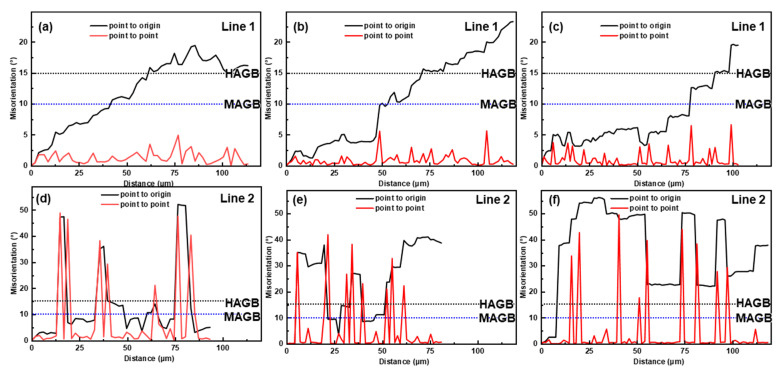
Cumulative and point-to-point misorientations along the lines of inside the deformed grains (**a**–**c**) and the DRX grains (**d**–**f**) at 0.1 s^−1^ at (**a**,**d**) 350 °C, (**b**,**e**) 400 °C, and (**c**,**f**) 450 °C.

The softening mechanisms were affected by temperature. Generally, Al alloys have a high stacking-fault energy (SFE) and are prone to DRV during hot deformation [29]. Meanwhile, a large number of precipitates in the matrix hindered the dislocation movement (Figure 8) [19]. Therefore, DRV was the dominant softening mechanism below 350 °C. Nevertheless, DRX occurred at 350 °C, albeit in a low proportion, and DRX became more and more pronounced with the increasing temperature. To distinguish the DRX mechanism, the MADs along specific paths in the IPF maps were analyzed, and the results are shown in Figure 10. In Figure 10, Line 1 corresponds to the MADs in the deformed grains, while Line 2 corresponds to the MADs along the necklace-distributed DRX grains.

Some original grains were segmented into substructures by the LAGBs, and HAGBs were detectable within the original grains (Figure 9a–c). As shown in Figure 10a–c, the point-to-point MADs were small, while the cumulative MADs exceeded 15°, indicating that the substructures gradually transformed into DRX grains via a progressive misorientation accumulation, namely CDRX [44,48]. The increase in the proportion of medium-angle grain boundaries (MAGBs, 10–15°) in Figure 9g–i also proved the occurrence of CDRX, because CDRX is accomplished by continuously absorbing dislocations at subgrain boundaries to increase their misorientation and induce subgrain rotation [25,49,50]. The occurrence of CDRX was unsurprising due to the rapid accumulation of strain gradients inside the grains [28]. Compared to the homogenized alloy, the matrix of the as-cast alloy was diluted because most of the solutes are stored in the eutectic T phase, which is more conducive to CDRX [17]. Nonetheless, CDRX was not fully developed due to inhibition by the dynamic precipitates [51]. On the other hand, some DRX grains nucleated at the triple junction or were distributed along the original grain boundaries in a necklace shape, which is typical of DDRX [29,48]. The MADs in Figure 10a–c show that there were HAGBs between the necklace-like distributed DRX grains, and the misorientations inside the DRX grains were weak, which further proves the occurrence of DDRX [48]. Although DDRX is usually observed in metals with low SFE, it may occur in Al-5Mg-3Zn-1Cu alloys because Mg solutes reduce the SFE of Al [52]. DDRX is accomplished through nucleation and growth. In Figure 9a–c, serrations and the bowing of the grain boundaries are universally observed, indicating that the nucleation mechanisms of DDRX were strain-induced boundary migration (SIBM) and grain boundary bulging [53]. During the hot deformation, the stress imbalance near the original grain boundaries led to the local bulging of these grain boundaries. Simultaneously, LAGBs were formed near the grain boundary with a high dislocation density, and these LAGBs separated the bulging. As the deformation progressed, these LAGBs continuously absorbed dislocations to increase their misorientation, and finally developed into HAGBs [20]. Additionally, some sporadic DRX grains with random orientations nucleated around the eutectic T phase, implying the activation of particle-stimulated nucleation (PSN) [54,55]. At 350 °C, the eutectic T phases had a poor deformability, so large strain gradients and orientation gradients were generated around them and provided favorable conditions for the DRX nucleation [35,36]. PSN provided supernumerary favorable DRX conditions that are not available for homogenized alloys [17]. As the temperature rose, the hindrance of the dislocation movement induced by the precipitates was weakened and the mobility of the grain boundaries was enhanced. Therefore, CDRX, DDRX, and PSN were simultaneously promoted, which manifested as an increased DRX fraction.

**Figure 11:**
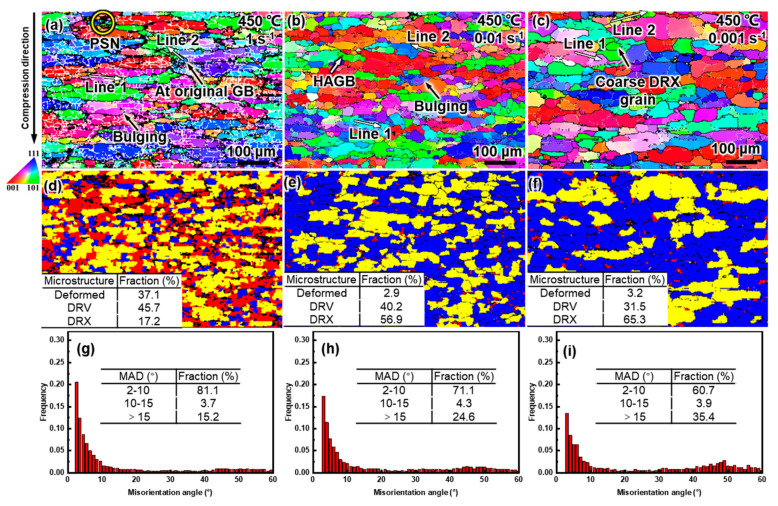
IPF maps (**a**–**c**), DRX maps (**d**–**f**), and misorientation angle distribution (**g**–**i**) of the alloy after compression at 450 °C at various strain rates: (**a**,**d**,**g**) 1 s^−1^, (**b**,**e**,**h**) 0.01 s^−1^, and (**c**,**f**,**i**) 0.001 s^−1^.

**Figure 12:**
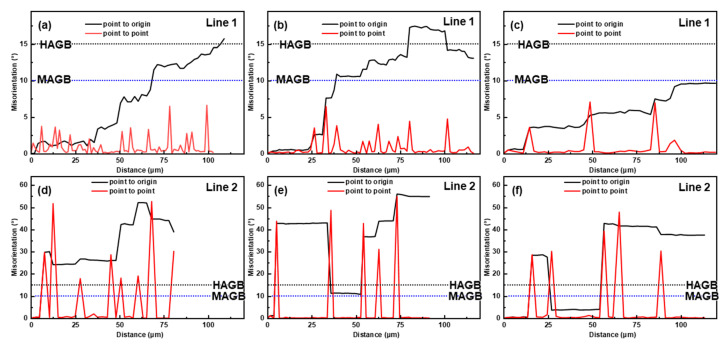
Cumulative and point-to-point misorientations along the lines of inside the deformed grains (**a**–**c**), and the DRX grains (**d**–**f**) at 450 °C at (**a**,**d**) 1 s^−1^, (**b**,**e**) 0.01 s^−1^, and (**c**,**f**) 0.001 s^−1^.

Figure 11 shows the grain structure of the alloy compressed at different strain rates at 450 °C. As shown in the IPF maps (Figure 11a–c), as the strain rate decreased from 1 to 0.001 s^−1^, the density of the LAGBs decreased continuously and the misorientation gradient also gradually eased. The proportion of microstructures in deformed and DRV states decreased, while that of DRX increased from 17.2% to 65.3% (Figure 11d–f), which was consistent with the evolution of MADs (Figure 11g–i). As the strain rate decreased, the dominant dynamic softening mechanism shifted from DRV to DRX. The DRX mechanism was also affected by the strain rate. From the MADs in Figure 12, it can be seen that, at 1 s^−1^, CDRX and DDRX still coexisted, and PSN can also be found in the IPF map (Figure 11a). When the strain rate was reduced to 0.01 s^−1^, the pinning effect of the precipitates was weakened, the multilateralization of the dislocation occurred, there was a transition from LAGBs to HAGBs, and the migration of the HAGBs became unimpeded, thus facilitating CDRX [50]. However, when the strain rate continued to decrease to 0.001 s^−1^, it was difficult for the cumulative misorientation inside the grains to reach MAGB (Figure 12c), and as a result, CDRX was suppressed, which is also confirmed by the reduced proportion of MAGBs in Figure 11g–i. DDRX was consistently promoted as the strain rate decreased throughout the entire range due to the enhanced grain boundary mobility and a further reduction in the SFE induced by the increased Mg solute in the matrix [52].

### 3.6. Conceptualization of Processing Map and Microstructure Evolution

**Figure 13:**
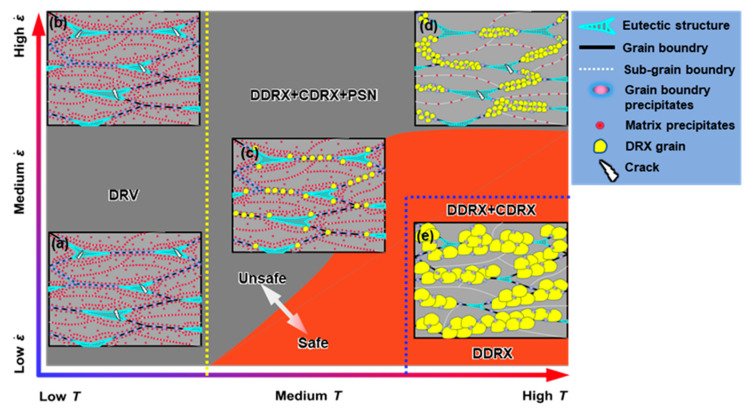
Schematic diagram epitomizing the microstructural features in the compressed alloy at (**a**) low T, low $\dot{\varepsilon }$; (**b**) low T, high $\dot{\varepsilon }$; (**c**) medium T, medium $\dot{\varepsilon }$; (**d**) high T, high $\dot{\varepsilon }$; and (**e**) high T, low $\dot{\varepsilon }$.

From Section 3.3 and Section 3.4, it can be found that the microstructure corresponded well with the processing maps. Figure 13 schematically shows the microstructural characteristics of the different regions in the processing map. At low temperatures (300 °C), deformation was unsafe. The eutectic T phase promoted cracking. The grains were in deformed or DRV states. Dynamic precipitates existed extensively within the grains and at the grain boundaries (Figure 13a,b). At medium temperatures (350 °C and 400 °C), the deformation was converted from unsafe to safe as the strain rate decreased. Fine DRX grains emerged and developed abundantly at the original grain boundaries, grain interiors, and the regions adjacent to the eutectic T phase through DDRX, CDRX, and PSN (Figure 13c), respectively. Both the intragranular and grain boundary precipitates were coarsened. At high temperatures (450 °C), instability occurred only at high strain rates. As the strain rate decreased, both the precipitates and eutectic T phases dissolved in large quantities, and the DRX grains grew significantly. DDRX was facilitated, while PSN was inhibited due to the dissolution of the eutectic T phase, and CDRX was first promoted and then inhibited.

## 4. Conclusions

The hot deformation characteristics of an as-cast Al-5.07Mg-3.01Zn-1.11Cu-0.01Ti alloy were evaluated using isothermal uniaxial compression in the range of 300–450 °C, 0.001–0.1 s^−1^, and the following conclusions were drawn:

(1) The rheological behavior presented characteristics of work hardening, followed by DRV/DRX, which was accurately described by the proposed strain-compensated Arrhenius-type constitutive model.

(2) A 3D processing map was established. Instability was mainly concentrated in regions with high strain rates or low temperatures, mainly occurring in the form of cracking. One workable domain was identified as 385–450 °C, 0.001 to 0.26 s^−1^.

(3) As the temperature rose, the dominant dynamic softening mechanism changed from DRV to DRX. The DRX mechanisms transformed from CDRX, DDRX, and PSN at 350 °C to CDRX and DDRX at 450 °C, 0.01 s^−1^, and eventually to DDRX at 450 °C, 0.001 s^−1^.

(4) The coarse eutectic T-Mg_32_(AlZnCu)_49_ phase facilitated DRX nucleation and did not trigger instability in the workable domain.

(5) The as-cast Al-5Mg-3Zn-1Cu alloy had a satisfactory hot workability.

## Data Availability

Not applicable.
